# Acquisition of antibodies to *Plasmodium falciparum* and *Plasmodium vivax* antigens in pregnant women living in a low malaria transmission area of Brazil

**DOI:** 10.1186/s12936-022-04402-4

**Published:** 2022-12-01

**Authors:** Meseret W. Kassa, Wina Hasang, André Barateiro, Timon Damelang, Jessica Brewster, Jamille G. Dombrowski, Rhea J. Longley, Amy W. Chung, Gerhard Wunderlich, Ivo Mueller, Elizabeth H. Aitken, Claudio R. F. Marinho, Stephen J. Rogerson

**Affiliations:** 1 Department of Medicine, University of Melbourne, The Peter Doherty Institute for Infection and Immunity, Level 5, 792 Elizabeth St, University of Melbourne, Melbourne, VIC 3000 Australia; 2 Department of Infectious Diseases, University of Melbourne, The Peter Doherty Institute for Infection and Immunity, Melbourne, VIC Australia; 3 Department of Parasitology, Institute of Biomedical Sciences, University of São Paulo, São Paulo, Brazil; 4 Department of Microbiology and Immunology, The Peter Doherty Institute for Infection and Immunity, University of Melbourne, University of Melbourne, Melbourne, VIC Australia; 5 Population Health and Immunity Division, Walter and Eliza Hall Institute of Medical Research, Melbourne, VIC Australia; 6 Department of Medical Biology, University of Melbourne, Melbourne, VIC Australia

**Keywords:** Pregnancy malaria, Antibody, *P. falciparum*, *P. vivax*, Low transmission

## Abstract

**Background:**

Pregnant women have increased susceptibility to *Plasmodium falciparum* malaria and acquire protective antibodies over successive pregnancies. Most studies that investigated malaria antibody responses in pregnant women are from high transmission areas in sub-Saharan Africa, while reports from Latin America are scarce and inconsistent. The present study sought to explore the development of antibodies against *P. falciparum* and *Plasmodium vivax* antigens in pregnant women living in a low transmission area in the Brazilian Amazon.

**Methods:**

In a prospective cohort study, plasma samples from 408 pregnant women (of whom 111 were infected with *P. falciparum*, 96 had infections with *P. falciparum* and *P. vivax*, and 201 had no *Plasmodium* infection) were used to measure antibody levels. Levels of IgG and opsonizing antibody to pregnancy-specific variant surface antigens (VSAs) on infected erythrocytes (IEs), 10 recombinant VAR2CSA Duffy binding like (DBL domains), 10 non-pregnancy-specific *P. falciparum* merozoite antigens, and 10 *P. vivax* antigens were measured by flow cytometry, ELISA, and multiplex assays. Antibody levels and seropositivity among the groups were compared.

**Results:**

Antibodies to VSAs on *P. falciparum* IEs were generally low but were higher in currently infected women and women with multiple *P. falciparum* episodes over pregnancy. Many women (21%-69%) had antibodies against each individual VAR2CSA DBL domain, and antibodies to DBLs correlated with each other (r ≥ 0.55, p < 0.0001), but not with antibody to VSA or history of infection. Infection with either malaria species was associated with higher seropositivity rate for antibodies against *P. vivax* proteins, adjusted odds ratios (95% CI) ranged from 5.6 (3.2, 9.7), p < 0.0001 for PVDBPII-Sal1 to 15.7 (8.3, 29.7), p < 0.0001 for PvTRAg_2.

**Conclusions:**

Pregnant Brazilian women had low levels of antibodies to pregnancy-specific VSAs that increased with exposure. They frequently recognized both VAR2CSA DBL domains and *P. vivax* antigens, but only the latter varied with infection. Apparent antibody prevalence is highly dependent on the assay platform used.

**Supplementary Information:**

The online version contains supplementary material available at 10.1186/s12936-022-04402-4.

## Background

Malaria during pregnancy remains a major public health problem in tropical and sub-tropical regions. In 2018, an estimated 11 million pregnant women were infected by *Plasmodium* in sub-Saharan Africa, which resulted in about 872,000 low birthweight deliveries [1]. Pregnant women have increased susceptibility to malaria, caused by placental accumulation of *Plasmodium falciparum* infected erythrocytes (IEs), leading to poor outcomes for both mother and infant, including maternal anaemia, miscarriage, stillbirth, low birthweight, and preterm birth [2]. The sequestration of IEs in the placenta is mediated by a *P. falciparum* erythrocyte membrane protein 1 (PfEMP1) family member, VAR2CSA [3], that binds to chondroitin sulfate A (CSA) [4], a glycosaminoglycan present on the placental syncytiotrophoblast surface. VAR2CSA is a large (350 kDa) protein containing six Duffy binding-like domains (DBL1–6) and interdomain (ID) regions [5]. Although *Plasmodium vivax* does not sequester in the placenta [6], it has also been associated with poor pregnancy outcomes [7–9]. The reasons for this may include systemic increases in inflammatory cytokines, such as tumour necrosis factor α and predisposition to severe anaemia (reviewed in [9]).

In high transmission areas, primigravidae are at highest risk of *P. falciparum* infection [2]. By contrast, Latin America is epidemiologically heterogeneous, *P. vivax* generally dominates, and the region is characterized by lower malaria transmission with some foci of higher endemicity and a prevalence of malaria in pregnancy generally below 10% [10, 11]; [12, 13]. In areas of lower transmission, all pregnant women may be susceptible to *P. falciparum* infection [10, 13–16].

With repeated exposure, pregnant women acquire antibodies against VAR2CSA that block adhesion of IEs to CSA [17, 18], which may improve pregnancy outcomes [19, 20]. Declining malaria transmission limits the development of these protective antibodies against pregnancy malaria [21, 22].

Studies of antibodies in pregnant women from Latin America have given inconsistent findings, and few have included antibody to *P. vivax* antigens. One group reported a high prevalence (> 60%) of antibodies to VAR2CSA recombinant protein in both pregnant women and in never-pregnant women, men, and children [23, 24]. They proposed that these might be cross-reactive antibodies induced by a specific epitope in the *P. vivax* Duffy-binding protein (PvDBP) [25, 26]. A second group confirmed a high prevalence of antibodies to recombinant VAR2CSA proteins expressed in baculovirus-transfected insect cells. By contrast, antibodies targeting native VAR2CSA on whole IEs and to recombinant VAR2CSA proteins expressed in Chinese hamster ovary (CHO) cells were rare [27]. The presence of antibody epitopes in the recombinant proteins produced in insect cells rather than induction of a cross-reactive antibody to VAR2CSA could explain the previous finding [27]. The lack of parity-dependent increases in antibody responses to VAR2CSA in Colombia [24, 26, 27] could reflect the low malaria transmission intensity or may indicate the presence of non-specific antibody responses.

To address some of these knowledge gaps and contradictory findings, the current study examined antibody to VAR2CSA expressed on whole IEs, including antibody that opsonizes IEs for phagocytosis, and measured IgG to recombinant VAR2CSA proteins, non-pregnancy malaria-associated *P. falciparum* antigens and *P. vivax* proteins in a cohort of pregnant Brazilian women. Correlations between different antibody responses were sought. The effects of gravidity and number of episodes of *Plasmodium* infection on the seroprevalence of these antibodies were analysed, and the effect of time since last *Plasmodium* infection on antibody levels was investigated.

## Methods

### Study site and participants

A prospective cohort study was conducted in the Amazonian region of the Alto do Juruá valley, Acre, Brazil. In a previous study in this location, 8.9% of pregnant women had microscopically documented malaria infection, over 60% of which were due to *P. vivax* [10]. In total, 600 pregnant women uninfected or infected by *Plasmodium* sp. were recruited during their first antenatal care visit and followed until delivery. Malaria was diagnosed from thin and thick blood smears by 2 experienced microscopists and confirmed by PET-PCR [15]. Isolates were not genotyped. Each woman was followed up by a trained nurse, which involved at least 2 domiciliary visits during the second and third trimester, to monitor the woman’s clinical state and to provide routine antenatal care. At the time of recruitment, data were collected on socioeconomic, clinical, and obstetric variables. During the domiciliary visits, clinical and obstetric data were obtained, and a peripheral blood sample was collected. Peripheral blood plasma collected at delivery was used in this study.

A total of 192 women who presented with other infections (cytomegalovirus, rubella, toxoplasmosis, herpes simplex virus, human immunodeficiency virus, hepatitis B virus, hepatitis C virus, syphilis, dengue, chikungunya, or Zika virus) were excluded [10]. Of the remaining 408 pregnant women who were studied, 111 were infected with *P. falciparum*, 96 had infections with *P. falciparum* and *P. vivax* concurrently or at different time points during the current pregnancy, and 201 had no *Plasmodium* infection during the pregnancy. Samples from women with *P. vivax* mono-infection were not available.

### Parasite culture

Two pregnancy malaria-associated *P. falciparum* lines, CS2 (which constitutively expresses VAR2CSA and binds to CSA) [28] and 3D7 (which was selected for CSA binding [29]) and a non-pregnancy associated *P. falciparum* line E8B-ICAM (which adheres to intercellular adhesion molecule (ICAM)–1 but not CSA) [30] were used. Parasites were cultured as previously described [31]. Cell cultures were synchronized as needed with 5% sorbitol [32] and subjected to gelatin flotation regularly to select knob-expressing IEs [33]. Adhesion to CSA and recognition by positive control sera were broadly similar for the 2 lines (not shown).

### Cell culture

Pro-monocytic THP-1 cells [34] were maintained in RPMI 1640 supplemented with 10% heat-inactivated fetal bovine serum (FBS), penicillin 1 unit/mL, streptomycin 1 µg/mL, glutamine 292 µg/mL and 25 mM HEPES buffer (all from Gibco™). The cells were maintained at a density of 2 × 10^5^ cells/ml in 75 cm^2^ cell culture flasks (Corning, 430641U) stored upright in a humidified 37°C incubator with 5% CO_2_, as previously described [35].

### Measuring IgG levels against VSAs

IgG antibody levels to pregnancy-specific VSAs on CS2 and 3D7 IEs were measured by flow cytometry as previously described [36]. In brief, test plasma was incubated with CS2 and 3D7 IEs (1:10) in duplicate, followed by incubation with polyclonal rabbit anti-human IgG (Dako A0424, 1:100 dilution), and with Alexa Fluor 647 donkey anti-rabbit (Invitrogen A32795, 1:500 dilution) containing 25 µg/ml of dihydroethidium (DHE), for 30 minutes in the dark. The cells were re-suspended in 2% paraformaldehyde (PFA) and acquired by flow cytometry (CytoFLEX S, Beckman Coulter) and analysed by FlowJo® software v10. The IgG level was expressed as relative geometric mean fluorescence intensity (MFI), represented as a percentage of the MFI of the positive control (pooled sera from *P. falciparum* infected Malawian multigravid women, after subtraction of the negative control (median MFI of sera from 18 malaria unexposed Melbourne donors; all controls were run individually, in the same experiments as test samples).

### Opsonic phagocytosis assay

The opsonic antibodies against CS2, 3D7 and E8B-ICAM were measured as previously described in [37]. In brief, purified trophozoite stage IEs stained with 25 µg/ml of DHE were resuspended at 1.67 × 10^7^/ml and opsonized with heat inactivated test plasma (1:10). Opsonized IEs were then incubated with THP-1 cells in a 5% CO_2_ humidified incubator at 37°C. Phagocytosis was stopped and non-phagocytosed IEs were lysed before cells were resuspended in 2% PFA. Test samples were then analysed by flow cytometry (CytoFLEX S, Beckman Coulter) and opsonizing antibody was determined as proportion of THP-1 cells positive for DHE and expressed as a relative percentage of positive control (pooled sera from *P. falciparum* infected Malawian multigravid women). Samples were defined as seropositive if the relative percentage was more than two standard deviations (SD) above the mean relative percentage of the unexposed controls (sera from 15 malaria naive Melbourne donors).

### Multiplex assay to measure antibodies to the recombinant VAR2CSA domains

Plasma IgG levels to VAR2CSA DBL domains (see Additional file 1) were measured using a multiplex assay as previously described in [38]. VAR2CSA DBL recombinant proteins were kind gifts from Joe Smith, Patrick Duffy, David Narum and Morten Nielsen. For antibody measurement, VAR2CSA DBL proteins coupled to Bio-Plex magnetic carboxylated microspheres (Bio-Rad) were incubated with test plasma (1:100 dilution in 1% bovine serum albumin (BSA) in phosphate-buffered saline (PBS)) in 96-well flat-bottom plates (Bio-Rad) on a shaker overnight at 4°C. Following incubation, plates were centrifuged and washed twice with PBS containing 0.05% (v/v) Tween-20 using a magnetic plate-washer (Bio-Plex Pro wash station). Then, anti-human IgG conjugated with phycoerythrin (PE) (SouthernBiotech, 9040-09, diluted to 1.3 µg/ml in 1% BSA in PBS) was added to each well and incubated for 2 hours on a plate shaker at room temperature. After washing with PBS containing 0.05% (v/v) Tween-20 and resuspending in sheath fluid (Life Technologies, MPXDF4PK), the plates were read on a Bio-Plex MAGPIX multiplex reader (Bio-Rad) and the MFI values were reported. Samples were designated antibody positive if the MFI was more than two SD above the mean MFI of the negative controls (sera from 8 malaria unexposed Melbourne donors).

### Multiplex assay to measure antibodies to the recombinant *P. vivax* proteins

IgG levels to *P. vivax* recombinant proteins (see Additional file 1) were measured using a multiplex assay described elsewhere [39]. The *P. vivax* proteins were kind gifts from Chetan Chitnis, Eizo Takashima and Takafumi Tsuboi. Protein-coupled microspheres were incubated with test plasma (1:100 dilution in PBS containing 1% BSA and 0.05% (v/v) Tween-20) in 96-well flat-bottom plates for 30 min at room temperature on a plate shaker. Following the incubation, microspheres were washed thrice with PBS containing 1% BSA and 0.05% (v/v) Tween-20. Then, anti-human IgG detector antibody conjugated with PE (Jackson ImmunoResearch (1 mg/ml), 1:100 dilution) was added to each well and incubated for 15 minutes at room temperature on a plate shaker.

After washing and resuspending in PBS containing 1% BSA and 0.05% (v/v) Tween-20, plates were read on a Bio-Plex MAGPIX multiplex reader (Bio-Rad) and the MFI values were reported. Samples were defined as seropositive if the MFI was more than two SD above the mean MFI of the negative controls (sera from 10 malaria unexposed Melbourne donors).

### IgG against non-pregnancy malaria-specific *P. falciparum* antigens

Antibody responses to schizont extract and *P. falciparum* MSP1-19 were measured by enzyme-linked immunosorbent assay (ELISA), as described elsewhere [40, 41]. PfMSP1-19 protein was cloned and expressed in *Escherichia coli* as previously described [40] and schizont extract was prepared from *P. falciparum* CS2-IEs [42]. Results were expressed relative to the Malawian positive control described above which had a value of 100 arbitrary units (AU). Samples were defined as antibody positive if the AU was more than two SD above the mean AU of the negative controls (8 malaria unexposed Melbourne donors for schizont extract and 10 malaria unexposed São Paulo donors for *Pf*MSP1-19).

### Statistical analysis

Demographic, clinical variables and antibody measurements were summarized with number and percentages for categorical variables and median and interquartile range (IQR) for continuous variables. Pearson chi-squared test was used to compare frequencies of seropositive individuals between groups, and Kruskal-Wallis rank sum tests followed by Dunn's multiple comparison test were used to compare the antibody levels amongst multiple groups. Spearman's rank test assessed the correlations between antibody to multiple antigens measured by different assays. Multiple logistic regression analysis was performed to determine the effect of *Plasmodium* infection and the number of episodes of *P. falciparum* infection on antibody seropositivity. Multivariate linear regression assessed the association between the time since the infection was diagnosed and antibody levels. P-values of less than 0.05 were considered statistically significant.

## Results

### Demographic and clinical characteristics of participants

Of the 408 pregnant women selected for this study, 207 (50.7%) had a *Plasmodium* spp. infection at some point in the current pregnancy. Of these, 111 had at least one *P. falciparum* infection, and 96 women had both *P. falciparum* and *P. vivax* infections. Maternal age, gravidity, and gestational age at delivery were similar across the three groups (Table 1). Women without parasitaemia episodes had higher hemoglobin levels at delivery (median (Inter quartile range (IQR)), 11.8 g/dL (11, 12.8)) compared to women with *P. falciparum* or with *P. falciparum* and *P. vivax* infections during pregnancy (median (IQR), 11.5 g/dL (10.7, 12.1), and 11.4 g/dl (10.5,12.2), respectively, p = 0.008). Birthweight tended to be lower in women with *Plasmodium* infection during pregnancy than in those without infection (p = 0.05) (Table 1).

**Table 1:** **Demographic and clinical characteristics of participants according to malaria infection status**

Characteristics		*P. falciparum* only (111)	*P. falciparum* and *P. vivax* (96)	Uninfected (201)	P-value
Maternal age at enrolment (years), median (IQR)		22 (17,28)	21 (18–27)	23 (19, 28)	0.22
Gravidity, n (%)					
Primigravidae		43 (39)	38 (40)	83 (41)	0.89
Multigravidae		68 (61)	58 (60)	118 (59)	
BMI at enrolment (kg/m^2^), median (IQR)		21 (20, 24)	22 (20, 25)	23 (20, 25)	**0.04**
Haemoglobin at delivery (g/dl), median (IQR)		11.5 (10.7, 12.1)	11.4 (10.5, 12.2)	11.8 (11, 12)	**0.008**
Gestational age at birth (weeks), median (IQR)		39 (38,40)	39 (38,40)	40 (39,41)	0.87
Birth weight (g), median (IQR)		3130 (2890, 3400)	3125 (2855, 3360)	3240 (2945, 3515)	0.05

Data are given as either number (n) (%) or median (Interquartile range (IQR)). *P. falciparum* represents individuals who had only *P. falciparum* infection (n = 111). *P. falciparum* and *P. vivax* represent women who had both infections with *P. falciparum* and *P. vivax* concurrently or at different time points during the current pregnancy (n = 96). Uninfected represents women with no *P. falciparum* or infection (n = 201). Pearson chi-squared tests were used to compare percentages, and Kruskal-Wallis rank sum tests were used to compare medians. P values that were less than 0.05 were indicated in bold.

*BMI* body mass index.

### Seroprevalence of antibodies to pregnancy-specific and non-pregnancy specific *Plasmodium *antigens

**Fig. 1:**
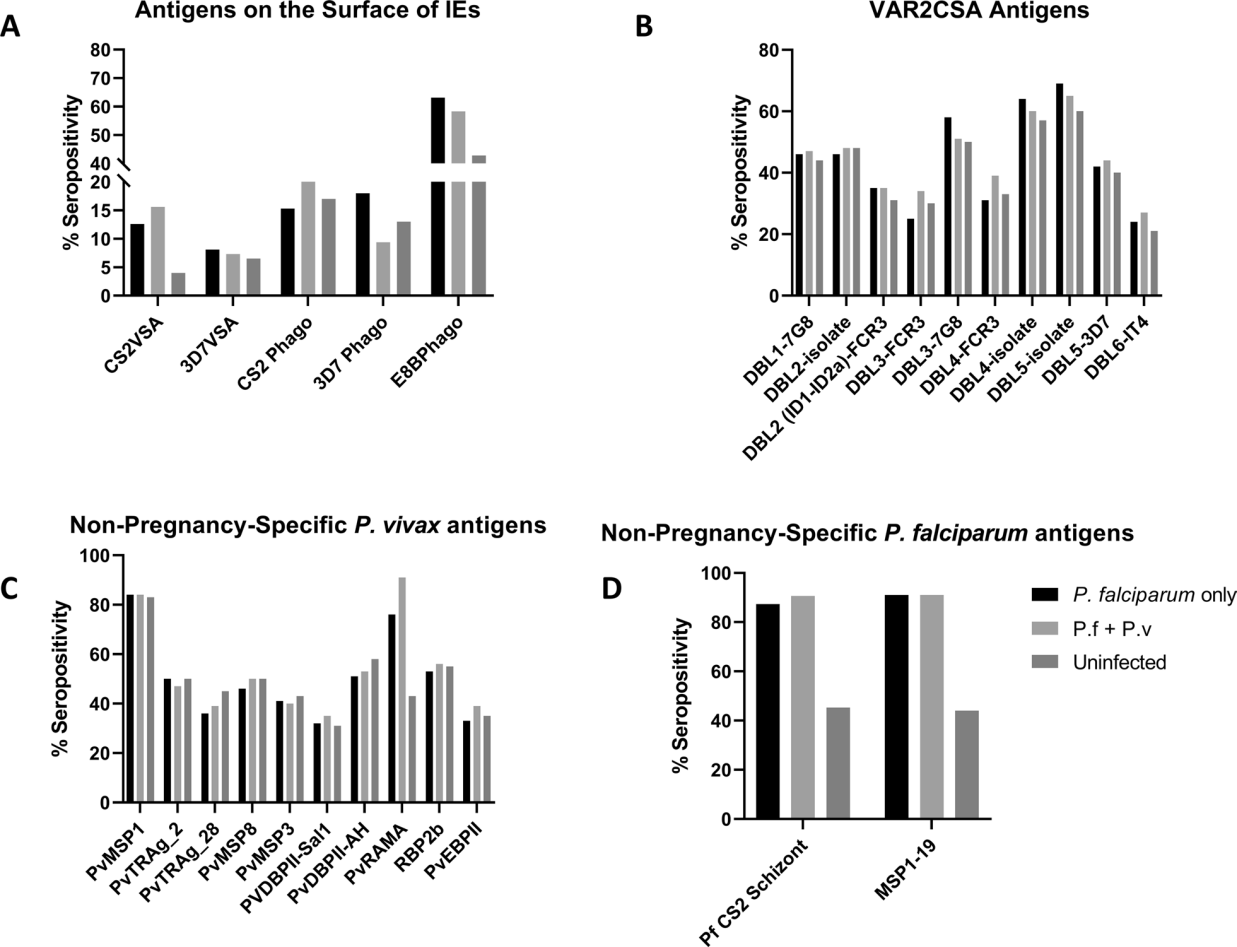
Seropositivity to pregnancy-specific and non-pregnancy specific***P. falciparum*** and ***P. vivax*** antigens in pregnant women living in the Brazilian Amazon. Data presented as the percentage of women seropositive to each antigen **A** Percentage seropositivity of total IgG and opsonic antibodies to antigens on the surface of intact IEs **B** Percentage seropositivity against recombinant VAR2CSA domains. **C** Percentage seropositivity to non-pregnancy specific *P. vivax* antigens **D** Percentage seropositivity to non-pregnancy specific *P. falciparum* antigens. VSA, variant surface antigens; DBL, Duffy binding like domain; ID, interdomain region, CS2 Phago, opsonic phagocytosis of CSA-binding CS2 IEs; 3D7 Phago, opsonic phagocytosis of CSA-binding 3D7 IEs; E8B Phago, opsonic phagocytosis of ICAM-1 binding E8B IEs; RAMA, Rhoptry-associated membrane antigen; MSP, merozoite surface protein; PvTRAg, tryptophan-rich antigen; PvDBP II-sal1, Duffy binding protein region II from ‘sal1’ strain; PvDBP II-AH, Duffy binding protein region II from ‘AH’ strain; RBP2b, reticulocyte binding protein 2b; PvEBP, erythrocyte-binding protein II

IgG antibodies to pregnancy-specific and non-pregnancy specific VSA on *P. falciparum* IEs were measured, and antibody seroprevalence was compared between women with *P. falciparum* infection, both *P. falciparum* and *P. vivax* infections, and without infection. When stratified by infection status 4–16% of women were seropositive for antibody to VSA on pregnancy-specific CSA binding CS2-IEs and 15–22% of women had opsonizing antibodies against pregnancy-specific VSA on CS2-IEs. By contrast, 43–63% of women had opsonizing antibodies to the non-pregnancy-specific VSAs of E8B-ICAM (Fig. 1A).

The proportion of women seropositive for antibodies to recombinant VAR2CSA DBL domains by multiplex assay varied from 60–69% (depending on exposure group) for DBL5-isolate to 21–27% (depending on exposure group) for DBL6-IT4 (Fig. 1B). Similarly, the proportions of women seropositive for antibodies to *P. vivax* proteins varied from 74–97% against Pv-MSP1-19 to 17–53% against PVDBPII-Sal1, (Fig. 1C). Around 90% of women with a history of previous *Plasmodium* infection at any time had antibodies to PfMSP1-19 and schizont extract (Fig. 1D).

### Comparison of antibody levels to VAR2CSA DBL domains and *P. vivax *proteins

**Fig. 2:**
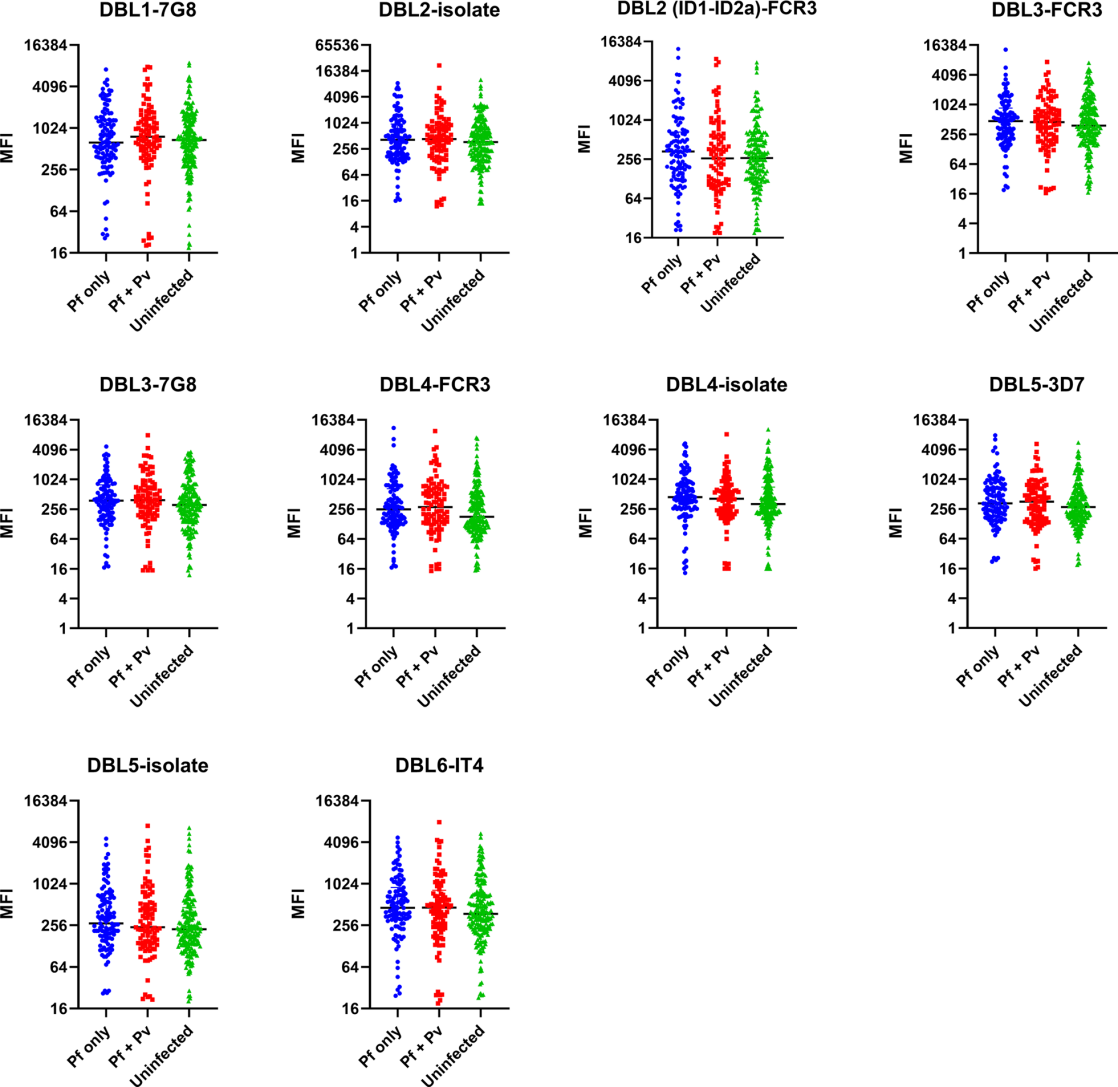
Levels of immunoglobulin G (IgG) antibody in pregnant women living in the Brazilian Amazon to recombinant VAR2CSA Duffy binding like (DBL) domains. The antibody levels between groups were compared by the Kruskal-Wallis test followed by Dunn's multiple comparison test. Values on the y-axis represent median IgG levels (expressed as MFI) against individual VAR2CSA domains. MFI, Median fluorescence index; ID, interdomain region. IT4, 3D7, 7G8 and FCR3 refer to the parasite strain sequence used for protein expression. *P ≤ 0.05

**Fig. 3:**
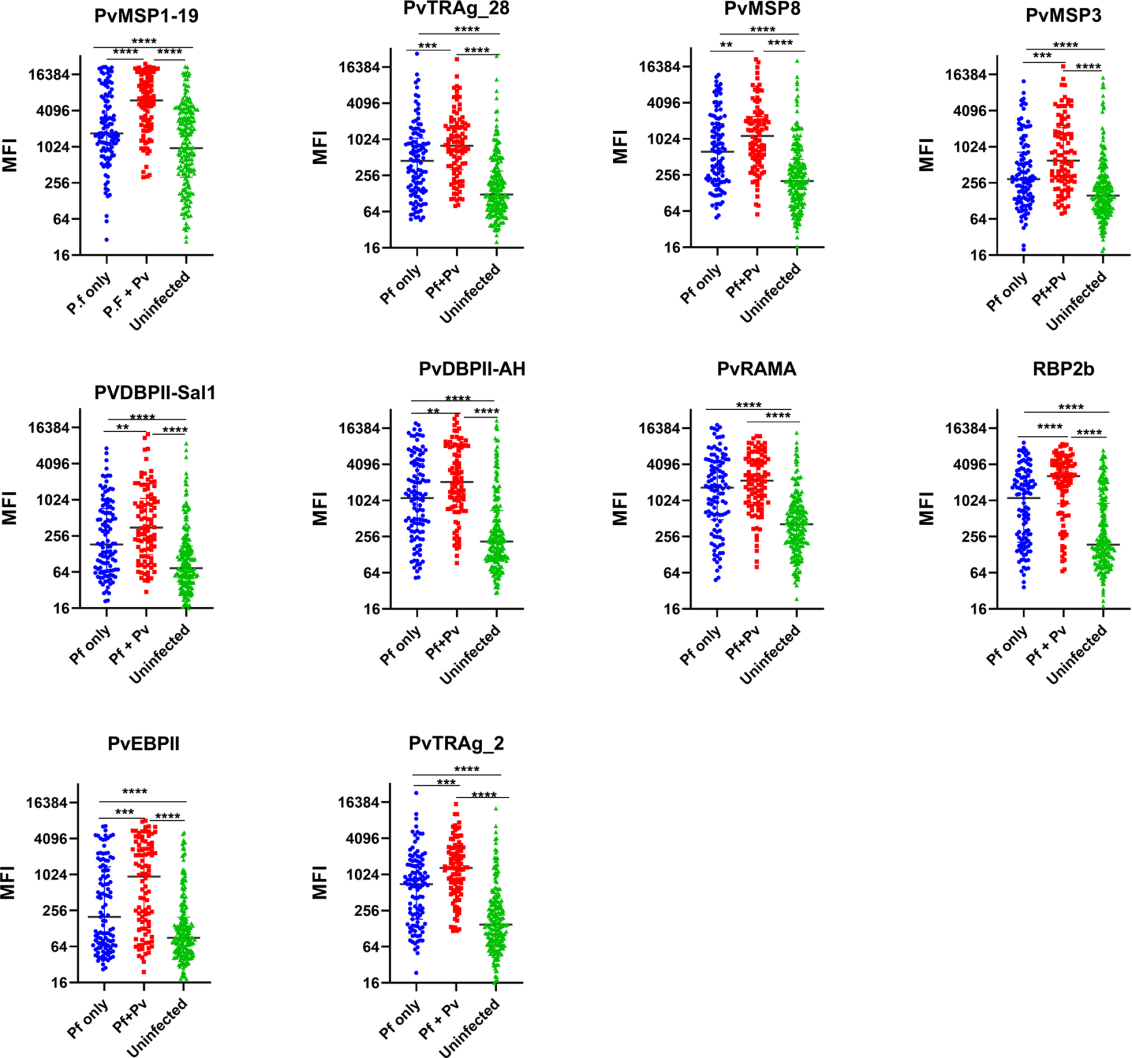
Levels of immunoglobulin G (IgG) antibody in pregnant women living in the Brazilian Amazon to recombinant ***P. vivax*** antigens. The antibody levels between groups were compared by the Kruskal-Wallis test followed by Dunn's multiple comparison test. Values on the y-axis represent mean IgG levels (expressed in MFI) against individual *P. vivax* antigens. MFI, Median fluorescence index; Pf, *Plasmodium falciparum*; Pv, *Plasmodium vivax*; RAMA, Rhoptry-associated membrane antigen; MSP, merozoite surface protein; PvTRAg, *P. vivax* tryptophan-rich antigen; PvEBP, *P. vivax* erythrocyte-binding protein; PvDBP II-sal1, *P. vivax* Duffy binding protein region II from ‘sal1’ strain; PvDBP II-AH, Duffy binding protein region II from ‘AH’ strain; RBP2b, reticulocyte binding protein 2b; **P ≤ 0.01, ***P ≤ 0.001, ****P ≤ 0.0001

Relative levels of IgG against *P. falciparum* VAR2CSA DBLs and *P. vivax* proteins were compared between women with *P. falciparum* only infection, both *P. falciparum* and *P. vivax infections*, or without malaria infection. The levels of IgG to VAR2CSA DBL domains were similar across the three groups (Fig. 2). However, IgG to *P. vivax* proteins varied significantly, being highest in women with both *P. falciparum* and *P. vivax* infections and lowest in uninfected women (Fig. 3). Interestingly, antibody levels to all *P. vivax* proteins were also higher in pregnant women with *P. falciparum* only than uninfected women (p < 0.0003) (Fig. 3).

### Antibody responses against VAR2CSA domains correlate with each other, but not with antibodies to surface antigens on intact IEs

**Fig. 4:**
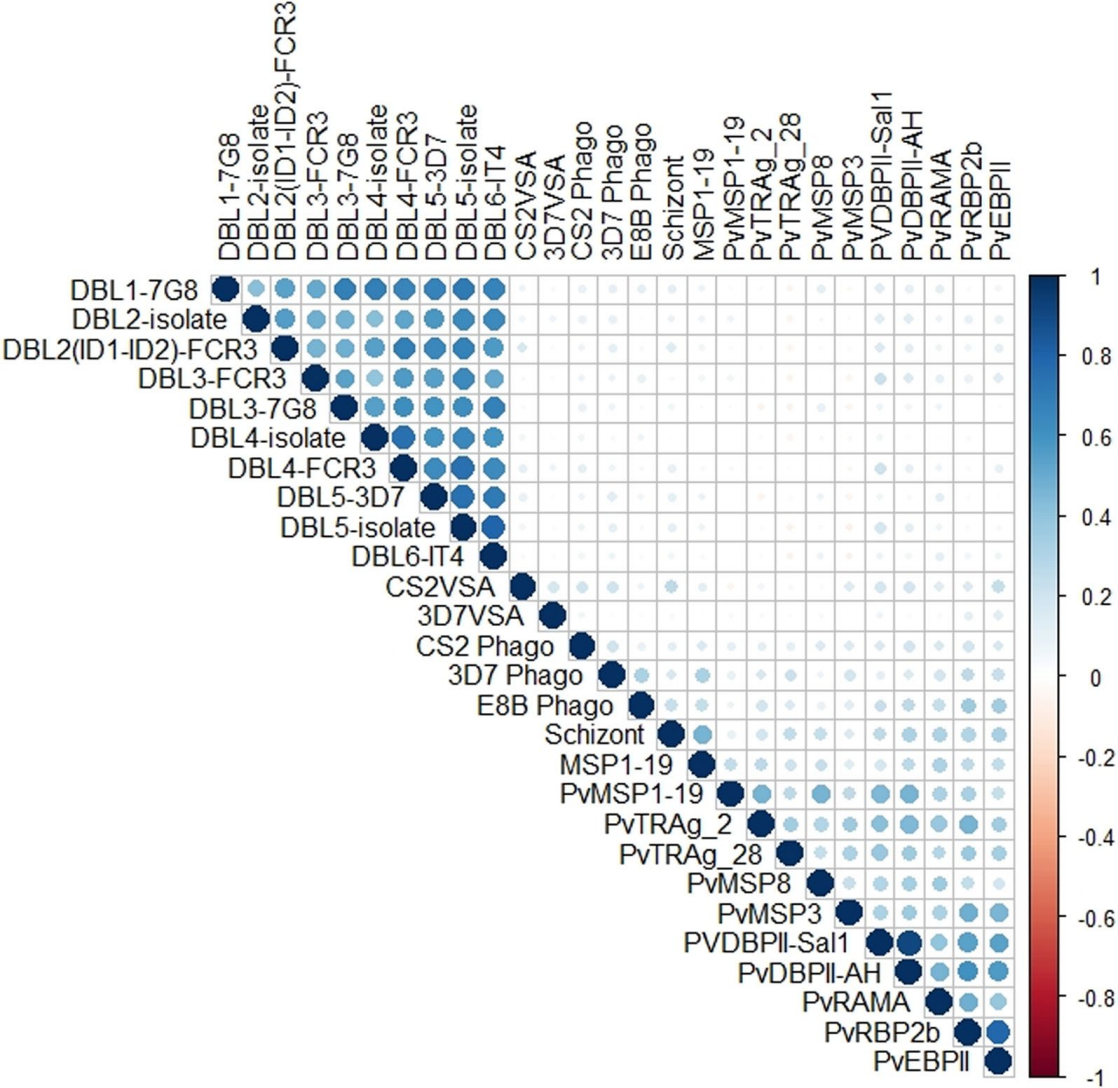
Heatmap of Spearman's correlation coefficients between 28 different antibody measurements. The color bar on the right indicates Spearman’s correlation coefficient from − 1 (negative correlation, red), no correlation (white) to 1 (positive correlation, blue). The intensity of the colors and the size of the circles indicate the correlation-values, with large, deeply colored blue or red circles indicate a relatively strong correlation, while small, lightly colored blue or red circles indicate a relatively weak correlation. VSA, variant surface antigens; DBL, Duffy binding like domain; ID, interdomain region; CS2 Phago, opsonic phagocytosis of CS2 infected erythrocytes (IEs); 3D7 Phago, opsonic phagocytosis of 3D7 IEs; E8B Phago, opsonic phagocytosis of E8B IEs; MSP, merozoite surface protein; Pv, *Plasmodium vivax*; RAMA, Rhoptry-associated membrane antigen; PvTRAg, *P. vivax* tryptophan-rich antigen; PvEBP, *P. vivax* erythrocyte-binding protein; PvDBP II-sal1, *P. vivax* Duffy binding protein region II from ‘sal1’ strain; PvDBP II-AH, Duffy binding protein region II from ‘AH’ strain; RBP2b, reticulocyte binding protein 2b

The correlation between the antibodies measured by different assays was investigated (Fig. 4). Antibodies to recombinant VAR2CSA DBL domains correlated significantly with each other (r ≥ 0.55, p < 0.0001) but not with antibodies to parasite antigens on surface of intact IEs (r ≤ 0.12, p > 0.06). Antibody to DBLs from different isolates (expressed in different expression systems) correlated with each other (r ≥ 0.62, p < 0.0001 for four pairs of similar constructs). Antibodies to recombinant *P. vivax* proteins also correlated significantly with each other (0.27 ≤ r ≤ 0.84, p < 0.0001) and showed weak to moderate correlation with antibodies to schizont extract (0.29 ≤ r ≤ 0.6, p < 0.0001) and *P. falciparum* MSP1-19 (0.35 ≤ r ≤ 0.47, p < 0.0001). Moreover, weak but significant correlation was observed between antibodies to *P. vivax* proteins and opsonic antibodies; CS2 phago (0.2 ≤ r ≤ 0.36, p < 0.0001), 3D7 phago (0.2 ≤ r ≤ 0.38, p < 0.0001), and E8B phago (0.1 ≤ r ≤ 0.31, p < 0.0001). Notably, the level of IgG to *P. vivax* proteins did not correlate well with the level of IgG against VAR2CSA DBLs (r ≤ 0.14, p ≥ 0.06).

### Gravidity and antibody to *P. falciparum* and *P. vivax* antigens

Next, the associations between gravidity and antibody seropositivity were investigated. Gravidity-dependent differences in seropositivity were observed for antibodies against VSA on CS2 IEs (p = 0.04). However, no gravidity dependent acquisition was observed for all other tested *P. falciparum* and *P. vivax* antigens (see Additional file 2).

### Association between malaria infection and seropositivity to *P.falciparum* antigens

To investigate whether malaria infection was a significant determinant of seropositivity, multivariate logistic regression was performed after adjusting for gravidity, maternal age, and body mass index (BMI) at enrollment. The participants were stratified into two groups: infected (women with *P. falciparum* only and both *P. falciparum* and *P. vivax* infections) and uninfected.

*Plasmodium*-infected women were more likely to have antibodies against pregnancy-specific VSAs on CS2 IEs (AOR (95% CI) = 4.1 (1.8, 9.1), p = 0.001) than uninfected women, and they were twice as likely to be seropositive for opsonic antibodies against E8B-ICAM IEs compared to their uninfected counterparts (AOR 2.1 (1.4, 3.1), p < 0.0001). However, the proportion of women with opsonic antibodies to VSA on placental binding IEs did not vary between infection groups (Table 2). There was no significant association between seropositivity to VAR2CSA DBL domains and infection, but malaria-infected women were more likely to be seropositive for antibodies to PfMSP1-19 and *P. falciparum* CS2 schizont extract than uninfected women (AORs 12.9 (7.4, 22.4), p < 0.0001) and 9.7 (5.8, 16.3), p < 0.0001, respectively) (Table 2).

**Table 2:** Associations between Plasmodium infection status and being seropositive for antibodies to *P. falciparum* antigens

Antigen	Infected (%) (n = 207)	Uninfected (%) (n = 201)	AOR (95% CI)	P-value
CS2VSA	14.0	4.0	4.1 (1.8, 9.1)	**0.001**
3D7VSA	7.3	6.5	1.2 (0.6, 2.6)	0.59
CS2 Phago	18.4	17.0	1.1 (0.7, 1.9)	0.69
3D7 Phago	14.0	13.0	1.1 (0.6, 1.9)	0.78
E8B Phago	60.9	42.8	2.1 (1.4, 3.1)	**< 0.0001**
DBL1-7G8	46.4	44.3	1.1 (0.74, 1.62)	0.65
DBL2-isolate	46.9	47.8	0.96 (.65, 1.42)	0.84
DBL2 (ID1-ID2)-FCR3	34.9	31.3	1.2 (.77, 1.77)	0.47
DBL3-FCR3	29.1	30.4	0.92 (0.60, 1.41)	0.71
DBL3-7G8	54.6	49.8	1.22 (0.83, 1.81)	0.30
DBL4-FCR3	34.8	32.8	1.08 (0.72, 1.64)	0.69
DBL4-isolate	61.8	56.7	1.24 (0.83, 1.84)	0.29
DBL5-isolate	42.5	39.8	1.1 (.74, 1.63)	0.65
DBL5-3D7	67.2	60.2	1.33 (0.89, 2.00)	0.17
DBL6-IT4	25.6	20.9	1.32 (0.83, 2.10)	0.24
Schizont	88.9	45.3	9.7 (5.8, 16.3)	**< 0.0001**
PfMSP1-19	91.8	44.3	12.9 (7.4, 22.4)	**< 0.0001**

Data presented as seropositivity percentage and odds ratio (95% confidence interval); Multivariate logistic regression analysis was performed to determine the effect of malaria infection on the antibody seropositivity in pregnant women with Plasmodium infection (*P. falciparum* only or both *P. falciparum* and *P. vivax* infections) and without Plasmodium infection. Analysis was adjusted for gravidity, body mass index at enrolment, and maternal age enrollment. AOR, Adjusted odds ratio; 95% CI, 95% confidence interval; Pf, *P. falciparum*; VSA, variant surface antigens; DBL, Duffy binding like; MSP, merozoite surface protein. P values that were less than 0.05 were indicated in bold

### Association between malaria infection and antibody seropositivity to *P. vivax* antigens

To investigate the association between *P. vivax* infection and seropositivity for antibodies against *P. vivax* proteins, women with both *P. falciparum* and *P. vivax* infections were compared to women without infection. Women with *Plasmodium* infection were more likely to be seropositive for antibodies to *P. vivax* proteins than uninfected women (AOR (95% CI) ranges from 5.6 (3.2, 9.7), p < 0.0001 for PVDBPII-Sal1 to 15.7 (8.3, 29.7), p < 0.0001 for PvTRAg_2; Table 3).

**Table 3:** Associations between *P. vivax* infection and being seropositive for antibodies to *P. vivax* antigens

Antigen	P. vivax (%)	Uninfected (%)	AOR (95% CI)	P-value
PvMSP1-19	96.9	74.1	10.7 (3.2, 35.4)	**< 0.0001**
PvTRAg_2	84.4	25.4	15.7 (8.3, 29.7)	**< 0.0001**
PvTRAg_28	69.8	21.9	8.2 (4.7, 14.3)	**< 0.0001**
PvMSP8	79.2	29.4	9.3 (5.2, 16.7)	**< 0.0001**
PvMSP3	70.8	25.4	7.0 (4.1, 12.1)	**< 0.0001**
PVDBPII-Sal1	47.3	16.9	5.6 (3.2, 9.7)	**< 0.0001**
PvDBPII-AH	78.7	30.4	14.6 (7.6, 28.2)	**< 0.0001**
PvRAMA	90.6	42.8	12.8 (6.1, 27.0)	**< 0.0001**
RBP2b	79.2	38.8	6.2 (3.5, 11.0)	**< 0.0001**
PvEBPII	59.4	17.9	6.9 (4.0, 12.0)	**< 0.0001**

Data presented as seropositivity percentage and odds ratio (95% confidence interval); Multivariate logistic regression analysis was performed to determine the effect of malaria infection on the antibody seropositivity in pregnant women with both *P. falciparum* and *P. vivax* infections and without plasmodium infection. Analysis was adjusted for gravidity, body mass index at enrolment, and maternal age enrollment. AOR, Adjusted odds ratio; 95% CI, 95% confidence interval; PV, *Plasmodium vivax*, RAMA, Rhoptry-associated membrane antigen; MSP, merozoite surface protein; PvTRAg, *P. vivax* tryptophan-rich antigen; PvEBP, *P. vivax* erythrocyte-binding protein; PvDBP II-sal1, *P. vivax* Duffy binding protein region II from ‘sal1’ strain; PvDBP II-AH, Duffy binding protein region II from ‘AH’ strain; RBP2b, reticulocyte binding protein 2b. P values that were less than 0.05 were designated in bold

### Influence of the number of *P*. *falciparum* episodes on the seropositivity

Antibody seropositivity was compared between women who had multiple episodes or a single episode of *P. falciparum* infection during the current pregnancy. Individuals with both *P. falciparum* and *P. vivax* infections or without *Plasmodium* infection were excluded from this analysis to investigate the influence of only *P. falciparum*. Out of 111 women infected with *P. falciparum*, 81 had a single infection, and 30 had multiple infections during the current pregnancy. In adjusted analyses, women who had multiple *P. falciparum* episodes during the current pregnancy were more likely to be seropositive for antibodies against VSAs on CS2 IEs (AOR (95% CI) = 4.0 (1.2, 13.6), p = 0.028) than women who had a single infection. The seropositivity against all other tested *P. falciparum* antigens did not differ significantly by the number of *P. falciparum* episodes (see Additional file 3).

### Association between time since infection and malaria antibody levels

Multivariate linear regression was performed to investigate whether antibody levels decline from the time the infection was diagnosed until delivery. Time since last infection date was available for 97 of 111 women infected with *P. falciparum* only and 51 of 96 women who had both *P. falciparum* and *P. vivax* infections. The time from last infection to delivery (in weeks) ranged from (median (IQR), 0.43 (0, 4.9) for *P. vivax* infection to 7 (0.57, 17.6) for *P. falciparum* infection. Generally, the levels of antibody against *P. falciparum* antigens were not significantly associated with the time since the *P. falciparum* infection was diagnosed. The exception was for antibodies to PfMSP1-19, which declined as time since the infection increased (coefficient (95% CI) = 1.5 (− 2.3, − 0.6), p = 0.001), suggesting that anti-PfMSP1-19 antibodies could be a marker of recent malaria exposure (see Additional file 4). Similarly, there were no significant associations between the time since *P. vivax* infection and the antibody levels against *P. falciparum* antigens, except for antibody to DBL3-7G8 which increased with increasing time.

Antibody levels to the *P. vivax* proteins tended to be negatively associated with the time since last *P. vivax* infection, but these trends did not reach statistical significance (p > 0.05). Similarly, no significant association was found between the antibody levels to *P. vivax* proteins and time since *P. falciparum* infection (see Additional file 4).

## Discussion

In areas of high malaria transmission, multigravid pregnant women acquire protective antibodies against pregnancy-associated malaria [43, 44]. In Latin America, transmission is often low, and *P. vivax* predominates, and recent studies have yielded conflicting data on the prevalence of antibody to VAR2CSA in pregnancy and non-pregnant populations [24, 26, 27]. The present study investigated the prevalence and levels of antibodies to pregnancy-associated and other malaria antigens in the Brazilian Amazon region, using a variety of assay formats and antibody targets including IEs, pregnancy-specific and non-pregnancy-specific *P. falciparum* recombinant proteins, and a panel of *P. vivax* antigens.

Antibody prevalence varied significantly depending on whether IE or recombinant antigens were used. When *P. falciparum* IEs were used, the prevalence of IgG antibodies and of opsonizing antibodies to pregnancy-specific VSAs was low, whereas most women had opsonizing antibodies to the VSA of a non-pregnancy-specific isolate. The low prevalence of antibody to pregnancy-specific VSAs may reflect low recent malaria transmission in the study area, while the higher recognition of E8B-ICAM is a product of higher lifetime exposure. Low levels and prevalence of antibodies to VAR2CSA antigens have been observed in settings of unstable malaria transmission [31], following a decline in malaria transmission, and in low transmission areas relative to high transmission areas of the same country [22, 45–48]. In contrast to pregnancy-associated IEs, recognition of recombinant VAR2CSA DBL proteins was common. Up to 69% of women recognized individual DBL proteins and recognition of individual DBLs was significantly correlated but seropositivity to recombinant DBL domains was not associated with *Plasmodium* infection. This was consistent with studies that reported a high prevalence of antibodies to VAR2CSA DBLs in Colombian pregnant women, children, and men with malaria, which did not differ between subjects with or without current *P. vivax* or *P. falciparum* infection [24, 27].

Although PfEMP1 is the predominant antigen on the surface of the IE [49], antibodies to recombinant VAR2CSA DBLs did not correlate with antibodies to antigens on the surface of VAR2CSA-expressing CS2 and 3D7CSA IEs. Previous studies have reported the existence of anti-recombinant VAR2CSA antibodies in non-pregnant populations [24, 26, 50–52], suggesting antibodies to recombinant VAR2CSA DBLs are not specific to pregnancy. This pattern might be related to the protein expression systems used. Post-translational glycosylation of recombinant proteins expressed in bacterial or insect cells can alter epitopes [53], and antibodies to full length VAR2CSA produced in baculovirus transfected insect cells were prevalent in Colombian pregnant women, men and children. Levels of antibodies to baculovirus-expressed VAR2CSA correlated strongly with antibodies to a similarly-expressed non-pregnancy related PfEMP1, whereas IgG towards native VAR2CSA antigen and VAR2CSA expressed in Chinese hamster ovary cells was restricted to pregnancy-associated malaria [27]. By contrast, and similar to the observations in the current study, antibodies that recognized pregnancy-specific IEs were uncommon, pregnancy-specific, and did not correlate with antibody to recombinant protein. This suggests that antibodies to recombinant VAR2CSA may recognize post-translational modifications that may be shared with non-*Plasmodium* proteins [27]. The VAR2CSA DBL proteins used in the present study were single domains expressed in bacterial, yeast, and insect cells (see Additional file 1). Levels of antibody did not to vary significantly with the expression system used and did not correlate with responses to IEs. They correlated moderately to strongly with one another irrespective of the expression system used. None of the proteins were expressed in Chinese hamster ovary cells [27]. Notwithstanding the high recognition of these proteins, it remains unclear whether recombinant VAR2CSA proteins are useful measures of exposure or immunity in South American populations [27].

In other studies from Colombia, antibodies to recombinant VAR2CSA domains were reported following exposure to *P. vivax* [26], and it has been suggested that heterologous immunity between *P. vivax* DBP and VAR2CSA could occur [25]. This study is only partly comparable with the earlier study, lacking non-pregnant individuals with malaria or individuals with *P. vivax* infections only. Antibody to different *P. vivax* proteins including Duffy binding protein correlated with one another, but there was no significant correlation between antibody to *P. vivax* antigens and VAR2CSA DBL domains. By contrast, in a cohort consisting of pregnant women from Brazil, Colombia, Guatemala, India, and Papua New Guinea correlations between antibodies to recombinant DBL proteins and recombinant *P. vivax* antigens were observed, although functional antibody that inhibited *P. vivax* Duffy binding protein interacting with its receptor did not correlate with other responses [54].

Pregnancy-specific antibodies often increase following infection and with increasing gravidity [44, 55]. In this cohort, only prevalence of IgG to CS2 IEs was higher in women with a history of *P. falciparum* infection compared to uninfected women and was positively associated with multiple *P. falciparum* infections. Antibodies to VAR2CSA DBLs did not differ in prevalence or magnitude between women with and without a history of *P. falciparum* infection during the index pregnancy. Similarly, only antibodies against pregnancy-specific VSAs on CS2 IEs increased with gravidity. The lack of matching associations with the 3D7CSA IEs may reflect higher VAR2CSA expression on CS2 than 3D7CSA [56, 57]. The parent isolate of CS2, It, originated in Brazil, and there could be a higher prevalence of CS2-like parasites in the population, but further studies of VAR2CSA genetic diversity [58] would be required. The present observations are consistent with the possibility that antibody to the recombinant DBLs are not pregnancy-specific, but antibody to IEs may be.

High proportions of women had antibodies to the *P. vivax* antigens tested, and antibody seropositivity and levels were higher in women with either *P. falciparum* only or both *P. falciparum* and *P. vivax* infections compared to uninfected women. The high seropositivity rate of antibodies to *P. vivax* antigens even among women without documented *P. vivax* during pregnancy may reflect the local predominance of *P. vivax* infection. Almost 70% of reported cases in Latin America are caused by *P. vivax* [59], and in the current cohort, *P. vivax* is the cause for 63.9% of infections [10]. In general, antibody levels to recombinant *P. vivax* antigens were higher in women with both *P. falciparum* and *P. vivax* infections than in those with *P. falciparum* only, possibly reflecting recent boosting as the *P. vivax* is the dominant species circulating in the area. The higher levels in women with *P. falciparum* than in uninfected women may reflect higher overall exposure to malaria, but are in contrast to findings from a multi country pregnancy cohort in which there was no evidence of higher antibody levels to *P. vivax* antigens in women with *P. falciparum* mono-infections compared to women without malaria infection at the time of sampling [54].

The relationship between the time since infection and the antibody level at delivery was also investigated. There were no significant associations between the time since either *P. falciparum* or *P. vivax* infection and antibody levels against pregnancy-specific *P. falciparum* antigens. On the other hand, antibodies to PfMSP1-19 decreased as the time since the last *P. falciparum* infection increased, suggesting that these antibody responses are relatively short-lived in pregnant women. This is broadly similar to a study from Thailand, which found that antibodies to merozoite antigens declined faster than antibody to VAR2CSA DBL5 in pregnant women [60], although the confidence intervals were large in the previous study. Furthermore, antibody levels to most *P. vivax* antigens also trended downwards as time since *P. vivax* infection increased, suggesting that repeated exposure may be required to maintain these antibody responses. The small sample size may have limited these findings, but they are in line with identification of specific proteins such as RBP2b (reticulocyte binding protein 2b) as markers of *P. vivax* infections in the prior 9 months [39], although these markers have not yet been validated in pregnant women.

Strengths of the study included the large number of women with documented *P. falciparum* and *P. vivax* infection, the longitudinal study design and the examination of antibody response using multiple experimental approaches. Weaknesses include the lack of sequential samples, which could have allowed more definitive conclusions regarding antibody decline following infections, and the relatively small number of women with multiple episodes of infection during pregnancy. Timing of infection during pregnancy relative to sampling varied quite widely. Infections were not genotyped, so it was not possible to confirm whether they were recrudescent or new infections. Inclusion of women with *P. vivax* alone would have added another dimension to the study.

## Conclusions

In summary, pregnant women in this region of Brazil had limited acquisition of antibodies to pregnancy-specific antigens on surface of whole IEs, despite having relatively high levels of antibodies to non-pregnancy associated *P. falciparum* antigens, recombinant DBL proteins, and *P. vivax* antigens. The lack of correlation between antibody to VAR2CSA DBL domains and antibody to surface antigen on IEs, or between antibody to VAR2CSA DBL domains and gravidity or malaria infection as markers of exposure, suggests they have limited utility in measuring immunity in this setting. Further studies are needed to define antibody responses that are robust markers of recent *P. falciparum* infection and/or may be correlates of protection from adverse pregnancy outcomes in low and unstable malaria transmission settings such as the Brazilian Amazon.

## Supplementary Information

Below is the link to the electronic supplementary material.

**Additional file 1: Table S1**List of P. falciparum VAR2CSA domains and P. vivax proteins tested in Luminex assays.

**Additional file 2: Table S2**The association between gravidity and antibody prevalence at delivery in women with and without history of malaria infection during current pregnancy.

**Additional file 3: Table S3**The association between the frequency of P. falciparum infections during current pregnancy and malaria antibody prevalence at delivery.

**Additional file 4: Table S4**The association between time since malaria infection was diagnosed and malaria antibody levels at delivery.

## Data Availability

The datasets used and/or analysed during the current study are available from the corresponding author on reasonable request.

## Abbreviations

AOR: Adjusted odds ratio
BMI: Body mass index
BSA: Bovine serum albumin
CHO: Chinese hamster ovary
CSA: Chondroitin sulfate A
DBL: Duffy binding like domain
DBP: Duffy-binding protein
DHE: Dihydroethidium
ELISA: Enzyme-linked immunosorbent assay
FBS: Fetal bovine serum
ICAM: Intercellular adhesion molecule
ID: Interdomain
IE: Infected erythrocytes
IQR: Inter quartile range
MFI: Mean fluorescence intensity
MSP: Merozoite surface protein
PBS: Phosphate-buffered saline
PE: Phycoerythrin
PFA: Paraformaldehyde
PfEMP1: *P. falciparum* erythrocyte membrane protein 1
PvDBP: *P. vivax* Duffy binding protein
PvEBP: *P. vivax* erythrocyte binding protein 2b
PvTRAg: *P. vivax* tryptophan-rich antigen
RAMA: Rhoptry-associated membrane antigen
RBP2b: Reticulocyte binding protein 2b
SD: Standard deviations
VSA: Variant surface antigens
