# Supplementary material for: Acquisition of antibodies to Plasmodium falciparum and Plasmodium vivax antigens in pregnant women living in a low malaria transmission area of Brazil
Source: Malar J. 2022 Dec 1;21:360. doi: 10.1186/s12936-022-04402-4 (PMC9714246; doi:10.1186/s12936-022-04402-4)
Supplement: Supplementary file 1 — Additional file 1: Table S1List of P. falciparum VAR2CSA domains and P. vivax proteins tested in Luminex assays. [file 12936_2022_4402_MOESM1_ESM.docx]

**Additional file 1. List of *P*. *falciparum* VAR2CSA domains and *P. vivax* proteins tested in Luminex assays**

| Protein | Expression system | Parasite species, strain or isolate (I) | Identifiers | Reference and source |
| --- | --- | --- | --- | --- |
| DBL1-7G8 | *Pichia pastoris* | *Pf,* 7G8 | MV-1398 | [1] |
| DBL2-isolate | *Escherichia coli* | *Pf, I-1010* | MV-1940 | [2] |
| DBL2 (ID1-ID2a)-FCR3 | *Escherichia coli* | *Pf,* FCR3 | MV-1942 | [2] |
| DBL3-FCR3 | Drosophila Schneider-2 cell | *Pf, FCR3* | MP1028 | [3] |
| DBL3-7G8 | *Pichia pastoris* | *Pf, 7G8* | MV-1914 | [1] |
| DBL4-isolate | *Escherichia coli* | *Pf, I- 0711* | MV-1700 | [2] |
| DBL4-FCR3 | *Escherichia coli* | *Pf, FCR3* | MP-2369 | [4] |
| DBL5-3D7 | *Pichia pastoris* | *Pf, 3D7* | 1218 | [1] |
| DBL5-isolate | *Escherichia coli* | *Pf, I-0466* | MV-1749 | [2] |
| DBL6-IT4 | *Pichia pastoris* | *Pf, IT4* | MV-1137 | [1] |
| Pv-MSP1-19 (PVX_099980) | Wheat germ cell-free | *PV* Sal 1 |  | [5] |
| PvTRAg_2 (PVX_096995) | Wheat germ cell-free | *PV* Sal 1 |  | [5, 6] |
| PvTRAg_28 (PVX_112670) | Wheat germ cell-free | *PV* Sal 1 |  | [5, 6] |
| PvMSP8 (PVX_097625) | Wheat germ cell-free | *PV* Sal 1 |  | [5] |
| PvMSP3 (PVX_097720) | Wheat germ cell-free | *PV* Sal 1 |  | [5] |
| PVDBPII-Sal1 (PVX_110810) | *Escherichia coli* | *PV* Sal 1 |  | [5] |
| PvDBPII-AH (AAY34130.1) | *Escherichia coli* | *PV* AH |  | [5] |
| PvRAMA (PVX_087885) | Wheat germ cell-free | *PV* Sal 1 |  | [5] |
| RBP2b (PVX_094255) | *Escherichia coli* | *PV* Sal 1 |  | [5] |
| PvEBPII (KMZ83376.1) | *Escherichia coli* | *PV* Brazil 1 |  | [5] |

*Pv,* P. vivax*, DBL, Duffy binding like domain; ID, interdomain region; RAMA, Rhoptry-associated membrane antigen; MSP, merozoite surface protein; PvTRAg, P. vivax tryptophan-rich antigen; PvEBP, P. vivax erythrocyte-binding protein; PvDBP II-sal1, P. vivax Duffy binding protein region II from ‘sal1’ strain; PvDBP II-AH, Duffy binding protein region II from ‘AH’ strain; RBP2b, reticulocyte binding protein 2b*

1. Avril M, Cartwright MM, Hathaway MJ, Smith JD: **Induction of strain-transcendent antibodies to placental-type isolates with VAR2CSA DBL3 or DBL5 recombinant proteins.** *Malar J* 2011, **10:**36.

2. Doritchamou JY, Herrera R, Aebig JA, Morrison R, Nguyen V, Reiter K, Shimp RL, MacDonald NJ, Narum DL, Fried M, Duffy PE: **VAR2CSA Domain-Specific Analysis of Naturally Acquired Functional Antibodies to Plasmodium falciparum Placental Malaria.** *J Infect Dis* 2016, **214:**577-586.

3. Nielsen MA, Pinto VV, Resende M, Dahlbäck M, Ditlev SB, Theander TG, Salanti A: **Induction of adhesion-inhibitory antibodies against placental Plasmodium falciparum parasites by using single domains of VAR2CSA.** *Infect Immun* 2009, **77:**2482-2487.

4. Fried M, Avril M, Chaturvedi R, Fernandez P, Lograsso J, Narum D, Nielsen MA, Oleinikov AV, Resende M, Salanti A, et al: **Multilaboratory approach to preclinical evaluation of vaccine immunogens for placental malaria.** *Infect Immun* 2013, **81:**487-495.

5. Longley RJ, White MT, Takashima E, Brewster J, Morita M, Harbers M, Obadia T, Robinson LJ, Matsuura F, Liu ZSJ, et al: **Development and validation of serological markers for detecting recent Plasmodium vivax infection.** *Nat Med* 2020, **26:**741-749.

6. Wang B, Lu F, Cheng Y, Chen JH, Jeon HY, Ha KS, Cao J, Nyunt MH, Han JH, Lee SK, et al: **Immunoprofiling of the tryptophan-rich antigen family in Plasmodium vivax.** *Infect Immun* 2015, **83:**3083-3095.

**References**
