# Supplementary material for: Acquisition of antibodies to Plasmodium falciparum and Plasmodium vivax antigens in pregnant women living in a low malaria transmission area of Brazil
Source: Malar J. 2022 Dec 1;21:360. doi: 10.1186/s12936-022-04402-4 (PMC9714246; doi:10.1186/s12936-022-04402-4)
Supplement: Supplementary file 2 — Additional file 2: Table S2The association between gravidity and antibody prevalence at delivery in women with and without history of malaria infection during current pregnancy. [file 12936_2022_4402_MOESM2_ESM.docx]

**Additional file 2: The association between gravidity and antibody prevalence at delivery in women with and without history of malaria infection during current pregnancy**

| Antigens | Infected | | | | Uninfected | | | |  |
| --- | --- | --- | --- | --- | --- | --- | --- | --- | --- |
|  | **Total (%)** | **PG (%)** | **MG (%)** | **P value^a^** | **Total (%)** | **PG (%)** | **MG (%)** | **P value^b^** | **P value^c^** |
| CS2VSA | 14.0 | 8.6 | 17.5 | 0.07 | 3.98 | 2.4 | 5.1 | 0.34 | **0.04** |
| 3D7VSA | 7.7 | 4.9 | 9.5 | 0.22 | 6.5 | 6.0 | 6.8 | 0.83 | 0.30 |
| CS2 Phago | 18.4 | 16.1 | 19.8 | 0.49 | 17.0 | 19.3 | 15.3 | 0.45 | 0.98 |
| 3D7 Phago | 14.0 | 13.6 | 14.3 | 0.89 | 12.9 | 8.4 | 16.1 | 0.11 | 0.23 |
| E8B Phago | 60.9 | 66.6 | 57.1 | 0.17 | 42.8 | 42.2 | 43.2 | 0.88 | 0.39 |
| DBL1-7G8 | 46.3 | 50.6 | 43.6 | 0.33 | 44.3 | 36.1 | 50.0 | 0.05 | 0.50 |
| DBL2-isolate | 46.9 | 46.9 | 46.8 | 0.99 | 47.8 | 46.9 | 48.3 | 0.85 | 0.90 |
| DBL2 (ID1-ID2)-FCR3 | 35.0 | 7.0 | 33.6 | 0.61 | 31.3 | 32.5 | 30.5 | 0.76 | 0.56 |
| DBL3-FCR3 | 29.1 | 30.9 | 28.0 | 0.66 | 30.4 | 32.5 | 28.8 | 0.57 | 0.48 |
| DBL3-7G8 | 54.7 | 56.8 | 53.2 | 0.61 | 49.8 | 50.6 | 49.2 | 0.84 | 0.62 |
| DBL4-FCR3 | 34.8 | 37.0 | 33.3 | 0.59 | 32.8 | 31.3 | 33.9 | 0.70 | 0.90 |
| DBL4-isolate | 61.8 | 66.8 | 58.7 | 0.25 | 56.7 | 55.4 | 57.6 | 0.76 | 0.56 |
| DBL5-isolate | 42.5 | 44.4 | 41.2 | 0.65 | 39.8 | 38.6 | 40.7 | 0.76 | 0.91 |
| DBL5-3D7 | 67.2 | 66.7 | 67.5 | 0.91 | 60.2 | 56.6 | 62.7 | 0.38 | 0.48 |
| DBL6-IT4 | 25.6 | 28.4 | 23.8 | 0.46 | 20.9 | 22.9 | 19.5 | 0.56 | 0.35 |
| Schizont | 88.9 | 91.4 | 87.3 | 0.37 | 45.3 | 46.9 | 44.1 | 0.68 | 0.41 |
| MSP1-19 | 90.8 | 88.9 | 92.1 | 0.44 | 44.3 | 45.8 | 43.2 | 0.72 | 0.94 |
| PvMSP1-19 | 92.8 | 90.1 | 94.4 | 0.24 | 74.1 | 71.1 | 76.3 | 0.41 | 0.19 |
| PvTRAg_2 | 72.5 | 75.3 | 70.6 | 0.46 | 25.4 | 20.5 | 28.8 | 0.18 | 0.69 |
| PvTRAg_28 | 59.9 | 67.9 | 54.8 | 0.06 | 21.9 | 21.7 | 22.0 | 0.95 | 0.16 |
| PvMSP8 | 68.1 | 71.6 | 65.9 | 0.39 | 29.4 | 26.5 | 31.4 | 0.46 | 0.92 |
| PvMSP3 | 57.5 | 60.5 | 55.6 | 0.48 | 25.4 | 20.5 | 28.8 | 0.18 | 0.73 |
| PVDBPII-Sal1 | 47.3 | 48.2 | 46.8 | 0.85 | 16.9 | 14.5 | 18.6 | 0.44 | 0.75 |
| PvDBPII-AH | 78.7 | 77.8 | 79.4 | 0.78 | 30.4 | 27.7 | 32.2 | 0.49 | 0.49 |
| PvRAMA | 82.6 | 86.4 | 80.2 | 0.25 | 42.8 | 38.5 | 45.7 | 0.31 | 0.92 |
| RBP2b | 70.5 | 72.8 | 69.1 | 0.56 | 38.8 | 42.2 | 36.4 | 0.41 | 0.32 |
| PvEBPII | 51.7 | 51.9 | 51.6 | 0.97 | 17.9 | 18.1 | 17.8 | 0.96 | 0.95 |

***a*** *Chi-square test: for the association between gravidity and antibody seropositivity in malaria-infected women*

***b*** *Chi-square test: for the association between gravidity and antibody seropositivity in malaria uninfected women*

***C*** *Cochran-Mantel-Haenszel test for the relationship between Ab positivity and gravidity after controlling for malaria status.*

*Data presented as frequency (proportion of seropositive women as a percentage). The threshold for seropositivity was the mean plus two standard deviations of the malaria unexposed Melbourne controls.* *PG, primigravidae; MG, multigravidae; VSA, variant surface antigens; DBL, Duffy binding like domain; ID, interdomain region, CS2 Phago, opsonic phagocytosis of CSA binding CS2 IEs; 3D7 Phago, opsonic phagocytosis of CSA binding 3D7CSA IEs; E8B Phago, opsonic phagocytosis of ICAM binding E8B IEs;* PV, Plasmodium vivax; *RAMA, Rhoptry-associated membrane antigen; MSP, merozoite surface protein; PvTRAg, P. vivax tryptophan-rich antigen; PvEBP, P. vivax erythrocyte-binding protein; PvDBP II-sal1, P. vivax Duffy binding protein region II from ‘sal1’ strain; PvDBP II-AH, Duffy binding protein region II from ‘AH’ strain; RBP2b, reticulocyte binding protein 2b. P values that were less than 0.05 were designated in bold.*
