# Supplementary material for: Acquisition of antibodies to Plasmodium falciparum and Plasmodium vivax antigens in pregnant women living in a low malaria transmission area of Brazil
Source: Malar J. 2022 Dec 1;21:360. doi: 10.1186/s12936-022-04402-4 (PMC9714246; doi:10.1186/s12936-022-04402-4)
Supplement: Supplementary file 3 — Additional file 3: Table S3The association between the frequency of P. falciparum infections during current pregnancy and malaria antibody prevalence at delivery. [file 12936_2022_4402_MOESM3_ESM.docx]

**Additional file 3.** **The association between the frequency of *P. falciparum* infections during current pregnancy and malaria antibody prevalence at delivery**

| Outcome | Infection | | AOR (95% CI) | P-value |
| --- | --- | --- | --- | --- |
|  | **Single (%) (n=81)** | **Multiple (%) (n=30)** |  |  |
| CS2VSA | 8.6 | 23.3 | 4.0 (1.2, 13.6) | **0.03** |
| 3D7VSA | 6.2 | 13.3 | 2.6 (0.6, 11.0) | 0.20 |
| CS2 Phago | 14.8 | 16.7 | 1.2 (0.3, 4.1) | 0.78 |
| 3D7 Phago | 14.8 | 26.7 | 1.8 (0.6, 5.2) | 0.28 |
| E8B Phago | 60.5 | 70.0 | 1.6 (0.6, 4.1) | 0.31 |
| DBL1-7G8 | 46.9 | 43.3 | 0.9 (0.4, 2.1) | 0.74 |
| DBL2-isolate | 44.4 | 50.0 | 1.3 (0.5, 3.0) | 0.61 |
| DBL2(ID1-ID2)-FCR3 | 39.5 | 20.7 | 0.4 (0.1, 1.1) | 0.07 |
| DBL3-FCR3 | 25.9 | 20.7 | 0.8 (0.3, 2.2) | 0.63 |
| DBL3-7G8 | 58.0 | 56.7 | 1.0 (0.4, 2.5) | 0.95 |
| DBL4-FCR3 | 30.8 | 33.3 | 1.2 (0.5, 2.9) | 0.75 |
| DBL4-isolate | 62.9 | 66.7 | 1.1 (0.5, 2.6) | 0.92 |
| DBL5-isolate | 38.3 | 50.0 | 1.7 (0.7, 4.1) | 0.23 |
| DBL5-3D7 | 70.4 | 66.7 | 0.9 (0.4, 2.3) | 0.85 |
| DBL6-IT4 | 24.7 | 23.3 | 0.9 (0.3, 2.6) | 0.91 |
| Schizont | 88.9 | 83.3 | 0.5 (0.2, 1.8) | 0.32 |
| PfMSP1-19 | 88.9 | 96.7 | 3.7 (0.4, 31.3) | 0.23 |

*Data presented as percentage (%) and odds ratio (95% confidence interval). Multivariate logistic regression analysis was performed to determine the effect of* P. falciparum *exposure on the antibody seropositivity in pregnant women with multiple exposures compared to a single exposure. Analysis was adjusted for gravidity and maternal age at enrollment. AOR, Adjusted odds ratio; 95% CI, 95% confidence interval; VSA, variant surface antigens; DBL, Duffy binding like domain; ID, interdomain region; CS2 Phago, opsonic phagocytosis of CSA binding CS2 IEs; 3D7 Phago, opsonic phagocytosis of CSA binding 3D7CSA IEs; E8B Phago, opsonic phagocytosis of ICAM binding E8B IEs; MSP, merozoite surface protein. P values that were less than 0.05 were designated in bold.*
