# Supplementary material for: Acquisition of antibodies to Plasmodium falciparum and Plasmodium vivax antigens in pregnant women living in a low malaria transmission area of Brazil
Source: Malar J. 2022 Dec 1;21:360. doi: 10.1186/s12936-022-04402-4 (PMC9714246; doi:10.1186/s12936-022-04402-4)
Supplement: Supplementary file 4 — Additional file 4: Table S4The association between time since malaria infection was diagnosed and malaria antibody levels at delivery. [file 12936_2022_4402_MOESM4_ESM.docx]

**Additional file 4.** The association between time since malaria infection was diagnosed and malaria antibody levels at delivery

| Outcome | Time since last *P. falciparum* infection (n = 97) | | Time since last *P. vivax* infection (n= 51) | |
| --- | --- | --- | --- | --- |
|  | **Coefficient (95% CI)** | **P value** | **Coefficient (95% CI)** | **P value** |
| CS2VSA | -10 (-112, 91) | 0.84 | -40 (-169, 89) | 0.54 |
| 3D7VSA | 40 (-7, 89) | 0.10 | -44 (-208, 120) | 0.59 |
| CS2 Phago | -0.2 (-0.1, 0.8) | 0.64 | -0.1 (-0.3, 0.2) | 0.53 |
| 3D7 Phago | -0.001 (-0.2, 0.2) | 0.10 | -0.03(-0.3, 0.2) | 0.81 |
| E8B Phago | 0.06 (-0.1, 0.25) | 0.52 | -0.18 (-0.5, 0.17) | 0.30 |
| DBL1-7G8 | -4.4 (-29, 21) | 0.73 | -3.8 (-80, 73) | 0.92 |
| DBL2-isolate | 3.4 (-16, 23) | 0.73 | 10 (-83, 104) | 0.83 |
| DBL2 (ID1-ID2)-FCR3 | -2.4 (-26, 21.5) | 0.85 | -10.4 (-99, 79) | 0.81 |
| DBL3-FCR3 | -6.7 (-33, 19) | 0.61 | -3.5 (-91, 84) | 0.94 |
| DBL3-7G8 | -0.9 (-22, 20) | 0.93 | **49 (15, 82.5)** | **0.01** |
| DBL4-FCR3 | 13 (-5.7, 32) | 0.17 | -8.3 (-54, 37) | 0.72 |
| DBL4-isolate | 5 (-18, 28) | 0.67 | -17 (-91, 56) | 0.64 |
| DBL5-isolate | 6.7 (-4.5, 18) | 0.24 | -0.7 (-54, 53) | 0.98 |
| DBL5-3D7 | -2 (-14, 10) | 0.74 | 3.2 (-23, 30) | 0.81 |
| DBL6-IT4 | 10 (-4, 25) | 0.16 | 3.2 (-38, 44) | 0.88 |
| Schizont | -0.6 (-0.1, 0.02) | 0.06 | -0.4 (-1, 0.3) | 0.29 |
| PfMSP1-19 | -1.5 (-2.3, -0.6) | **0.001** | -0.4 (-1.9, 1.1) | 0.57 |
| PvMSP1-19 | 20 (-118, 160) | 0.77 | 36 (-299, 371) | 0.83 |
| PvTRAg_2 | -43 (-101, 45) | 0.15 | -84 (-220, 51) | 0.21 |
| PvTRAg_28 | -12 (-76, 52) | 0.71 | -50 (-230, 129) | 0.58 |
| PvMSP8 | -5.9 (-67, 55) | 0.85 | 4.3 (-143, 152) | 0.95 |
| PvMSP3 | -4.3 (-31, 22.5) | 0.75 | -111 (-302, 79) | 0.24 |
| PVDBPII-Sal1 | 4.3 (-16, 25) | 0.69 | -67 (-192, 59) | 0.29 |
| PvDBPII-AH | -34 (-121, 53) | 0.44 | -161 (-470, 147) | 0.29 |
| PvRAMA | -50 (-131, 31) | 0.22 | -101 (-246, 45) | 0.17 |
| RBP2b | 21.4 (-38, 80) | 0.48 | -144 (-296, 7.5) | 0.06 |
| PvEBPII | 0.7 (-34, 35) | 0.97 | -38 (-149, 74.1) | 0.50 |

*Data presented as a coefficient and 95% confidence interval. P values were determined by multivariate linear regression analysis. Models were adjusted for all relevant and available covariates that could confound the association between the time since the infection was diagnosed and antibody levels. The selected covariates were* *gravidity, maternal age at enrollment, and BMI at enrollment. 95% CI, 95% confidence interval; VSA, variant surface antigens; DBL, Duffy binding like domain; ID, interdomain region; CS2 Phago, opsonic phagocytosis of CSA binding CS2 IEs; 3D7 Phago, opsonic phagocytosis of CSA binding 3D7CSA IEs; E8B Phago, opsonic phagocytosis of ICAM binding E8B IEs;* PV, Plasmodium vivax; *RAMA,* *Rhoptry-associated membrane antigen; MSP, merozoite surface protein; PvTRAg, P. vivax tryptophan- rich antigen; PvEBP, P. vivax erythrocyte-binding protein; PvDBP II-sal1, P. vivax Duffy binding protein region II from ‘sal1’ strain; PvDBP II-AH, Duffy binding protein region II from ‘AH’ strain; RBP2b, reticulocyte binding protein 2b. P values that were less than 0.05 were designated in bold.*
